# Supplementary figures and images for: A novel task to investigate vibrotactile detection in mice
Source: PLoS One. 2023 Apr 20;18(4):e0284735. doi: 10.1371/journal.pone.0284735 (PMC10118142; doi:10.1371/journal.pone.0284735)

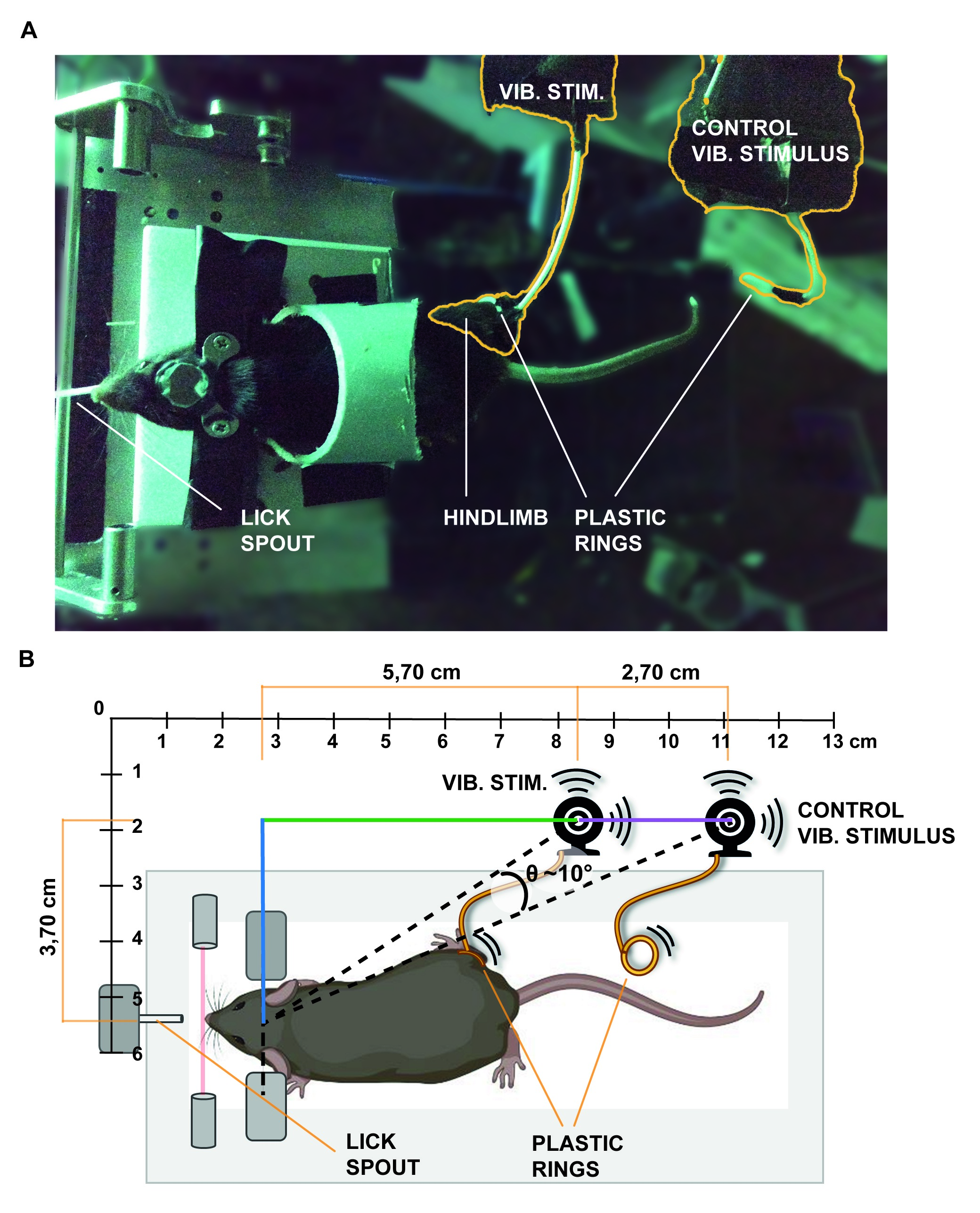

Supplement: S1 Fig — (A) Photo showing a top view of the experimental setup, with highlighted the positions of the vibratory motors and of the rigid plastic rings to deliver the vibration. (B) Schematic diagram of the experimental setup indicating the relative position of the mouse and of the vibratory motors. Positions are calculated relative to the midline between the ears. Reprinted from Biorender under a CC BY license, with permission from Biorender, original copyright 2023. (TIF) [file pone.0284735.s001.tif]
